# Does Total Body Irradiation Have a Favorable Impact on Thrombocyte Engraftment as per Neutrophil Engraftment in Allogeneic Stem Cell Transplantation?

**DOI:** 10.7759/cureus.19462

**Published:** 2021-11-11

**Authors:** Bahar Uncu Ulu, Tuğçe Nur Yiğenoğlu, Derya Şahin, Semih Başcı, Dicle İskender, Yasemin Adaş, Ebru Atasever Akkaş, Tuba Hacıbekiroğlu, Merih Kızıl çakar, Mehmet Sinan DAL, Fevzi Altuntaş

**Affiliations:** 1 Department of Hematology and Bone Marrow Transplantation, Dr. Abdurrahman Yurtaslan Ankara Oncology Training and Research Hospital, University of Health Sciences, Ankara, TUR; 2 Radiation Oncology, Dr. Abdurrahman Yurtaslan Ankara Oncology Training and Research Hospital, University of Health Sciences, Ankara, TUR; 3 Hematology, Sakarya University Hospital, Sakarya, TUR; 4 Department of Hematology, Ankara Yıldırım Beyazıt University, School of Medicine, Ankara, TUR

**Keywords:** acute leukemia, hematopoietic stem cell transplantation, neutrophil engraftment, thrombocyte engraftment, total body irradiation

## Abstract

Introduction: In this study, we aim to analyze the effect of total body irradiation (TBI) on neutrophil and thrombocyte engraftment durations in acute leukemia (AL) patients who achieved allogeneic hematopoietic stem cell transplantation (Allo-SCT) at our center.

Methods: The data of 193 acute leukemia patients who were performed Allo-SCT from matched-related donors were analyzed retrospectively.

Results: Thrombocyte engraftment duration was statistically shorter (12 days) in acute lymphoblastic leukemia (ALL) patients who received TBI-based conditioning when compared to ALL patients who received non-TBI-based conditioning (14 days; p=0.037). On the other hand, no statistically significant difference was observed between acute leukemia patients who received TBI or non-TBI-based conditioning regarding neutrophil engraftment duration.

Conclusion: We found that TBI had a favorable impact on thrombocyte engraftment (TE) rather than neutrophil engraftment (NE) in Allo-SCT in patients with acute leukemia. TBI might have an impact on the engraftment of thrombocytes as per than neutrophils may be attributed to immune mechanisms and microenvironment in the patient’s bone marrow (BM).

## Introduction

Acute leukemias (AL) are hematological malignancies characterized by abnormal proliferation of blasts caused by hematopoietic myeloid or lymphoid precursors or both. In adults, the most frequently seen AL type is acute myeloid leukemia (AML) and it has an incidence of 5-8/100.000 [1,2]. Acute lymphoblastic leukemia (ALL) is the most frequently seen AL type in children. In adults, it has an incidence of 1.28/100.000 and it is less commonly observed compared to AML [3].

Allogeneic hematopoietic stem cell transplantation (Allo-SCT) is used as a curative treatment in patients with relapsed, refractory, or high-risk acute leukemia [4-6]. After the infusion, hematopoietic stem cells (HSCs) settle in the bone marrow (BM) niche where they can find optimal conditions to survive and proliferate [7]. The goal for successful engraftment is to provide long-term effective hematopoiesis and produce all hematopoietic cell subsets [8]. Previous studies revealed that graft failure is associated with worse survival compared to sustained engraftment of donor cells [8,9]. Several risk factors have been identified for graft failure that may be related to the patient, the donor, or the transplant procedure [10].

Total body irradiation (TBI) has been used in Allo-SCT for almost 50 years [11]. It shows that TBI could eradicate resistant malignant cells effectively in the bone marrow and reduce tumor burden even at extranodal sites such as the central nervous system and testis where chemotherapy is relatively ineffective [12]. Total body irradiation damages HSCs in the BM and provides elimination of lymphocytes to prevent the donor’s HSCs rejection and ensure the physical space for hematopoietic cell engraftment [13]. However, TBI is associated with a wide variety of side effects, including heart, lung, and kidney complications, cataracts, new malignancies, and an increased risk of developing endocrinopathies [14,15]. On the other hand, TBI has immunosuppressive effects and may prevent graft rejection [16]. In recent years, the strategies of HSCs transplantation have changed, including the intensity of the conditioning regimens, prophylaxis of graft-versus-host disease (GVHD), donor selection, prophylactic strategies during febrile neutropenia, and supportive care. Therefore, engraftment success seems to be affected by changing strategies.

As there is an association between engraftment and survival, it is very important to find out all the factors related to engraftment failure. In this study, we aimed to analyze the effect of TBI on neutrophil and thrombocyte engraftment durations in AL patients who were performed Allo-SCT at our center.

## Materials and methods

Patients

The data of AL patients who were performed Allo-SCT from their Human leukocyte antigen (HLA) - 9/10 or 10/10 matched related donors between December 2009 and December 2018 - were analyzed retrospectively. The patients whose AL were diagnosed with the examination of the morphological findings of BM aspirates and flow cytometry or immune histochemical analysis, and who were over the age of 18 years were included in the study. The data regarding the gender, age, AL type, conditioning regimen, donor characteristics, and engraftment durations were retrospectively analyzed. HLA evaluation was performed with a high-resolution method of HLA-A, HLA-B, HLA-C, and HLA-DRB1. The matched donor was defined as the donor matched at high resolution for HLA-A, HLA-B, HLA-C, and HLA-DRB1 (HLA-10/10 matched or 9/10 matched). Peripheral blood (PB) derived HSCs were used in all Allo-SCTs. Haploidentical transplantations and transplantations from unrelated donors were excluded.

Engraftment durations

The duration between the date of stem cell infusion and the date when the absolute neutrophil count (ANC) was >500/mm^3^ and thrombocyte count was >20,000/mm^3^ for three consecutive days without any support were defined as neutrophil engraftment (NE) and thrombocyte engraftment (TE), retrospectively [17].

Total body irradiation

Patients received radiation therapy with bilateral parallel opposing fields and 6-MV photons in ELECTA Synergy Platform Linear Accelerator. Patients were immobilized in the supine position. The manual treatment planning was delivered; 372 cm source-to-axis distance and 40×40 cm^2^ field size with 90˚ gantry and 45˚ collimator angle was used for treatment. To measure the radiation doses, Mosfet in vitro dosimetry was applied to the head, neck, chest, and umbilicus. Tissue equivalent material was used to decrease the dose of lung, neck, and head to the planning region. One fraction of the treatment was approximately 30 minutes, and a minimum of a six-hour break was given between two fractions. Eight gray TBI was given in twice-daily fraction for two days (2×2 Gray/day for two days) and 12 gray TBI was given twice-daily fraction for three days (2×2 Gray/day for three days).

Statistical analyses

For statistical analysis, IBM SPSS Statistics (version 21, IBM Corp., Armonk, NY) software was used. Categorical data were expressed as a ratio, and numerical data were expressed as a median and a mean ± standard deviation. The differences between neutrophil and platelet engraftment times across age groups were examined by the non-parametric Mann-Whitney U test.

Ethics

The trial was conducted following the guidelines of the Declaration of Helsinki. The Ethical Committee approved the study protocol of the Coordinating Center of Dr. Abdurrahman Yurtaslan Ankara Oncology Training and Research Hospital (2020-06/663).

## Results

One hundred and ninety-three patients with AL were included in the study. Seventy-two patients received TBI-based conditioning regimens whereas 121 patients received non-TBI-based conditioning regimens. There were 106 AML patients and 87 ALL patients. Nearly 83% of the patients received TBI-based conditioning in ALL patients whereas 17% of the ALL patients had non-TBI-based conditioning regimens. All AML patients received a non-TBI-based conditioning regimen, none of the AML patients received a TBI-based regimen. Characteristics of ALL and AML patients were given in Table 1. When the gender ratio and the median ages were compared, ALL and AML groups were similarly distributed (p=0.8 and p=0.7, respectively). Regarding infused CD34+ cell numbers, there was no statistical difference between ALL and AML patients (p=0.4). No statistical difference was found between AML and ALL patients regarding median NE duration (15 days, p=0.9). Thrombocyte engraftment duration was statistically significantly shorter in ALL patients (12 days) compared to AML patients (14 days; p=0.001).

**Table 1:** Characteristics of acute leukemia patients ALL: acute lymphoblastic leukemia; AML: acute myeloid leukemia; CY-BU: cyclophosphamide busulfan; FLU-BU-ATG: fludarabine busulfan antitimosit globulin; CY-TBI: cyclophosphamide total body irradiation; FLU-ATG-TBI: fludarabine antitimosit globulin total body irradiation; BU-FLU-ATG: busulfan fludarabine antitimosit globulin; CY-BU-ATG: cyclophosphamide busulfan antitimosit globulin, N/A: not applicable

	ALL (n=87)	AML (n=106)	p-value
Gender (female/male; n)	31/56	36/70	0.8
Median age (years)	32 (18–59)	34 (18–62)	0.7
Conditioning regimen			
Non-TBI-based (n=121)	CY-BU (n=5)	CY-BU-ATG (n=64)	N/A
	FLU-BU-ATG (n=10)	BU-FLU-ATG (n=42)	
TBI-based (n=72)	CY-TBI (12 Gy) (n=55)		N/A
	CY-TBI (8 Gy) (n=7)		
	FLU-ATG-TBI (12 Gy) (n=10)		
Median CD34+ cell number infused	7.2×10^6^/kg (2.94–14.86)	6.8×10^6^/kg (2–10.28)	0.4
Median neutrophil engraftment duration (days)	15 (10–30)	15 (10–43)	0.9
Median thrombocyte engraftment duration (days)	12 (9–27)	14 (10–84)	0.001

**Figure 1:**
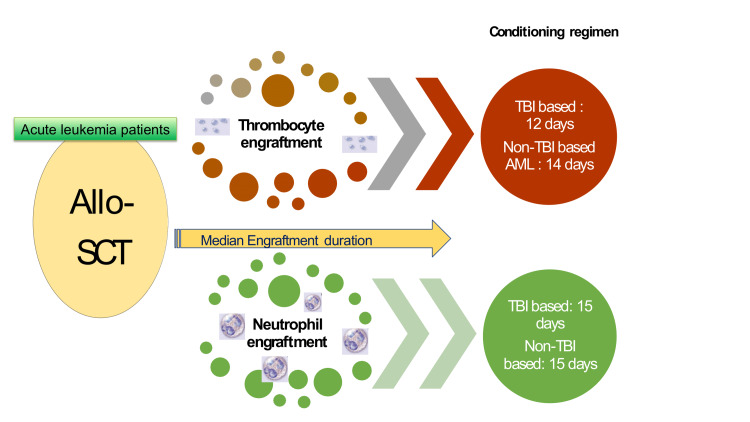
Summary of engraftment durations Allo-SCT: allogeneic hematopoietic stem cell transplantation, TBI: total body irradiation

The median engraftment durations in all patients according to their conditioning regimens were given in Table 2 and Figure 1. Thrombocyte engraftment duration was significantly shorter in all cohorts who received TBI-based conditioning (12 days; range 9-27 days) comparing non-TBI-based conditioning (14 days; range 10-84; p= 0.007), on the other hand, no statistically significant difference was observed in all cohort who received non-TBI-based conditioning (15 days, range 10-43 days) or TBI-based conditioning regarding NE duration (15 days, range 10-30 days; p=0.03). Since the number of ALL patients who received 8 Gy TBI was lower than those who received 12 Gy TBI, a comparison was not made between the two groups.

**Table 2:** Median engraftment durations in all cohorts according to their conditioning regimens TBI: total body irradiation

	All patients		p-value
Conditioning regimen	TBI based	Non-TBI based	
Median neutrophil engraftment duration (days)	15 (10–30)	15 (10–43)	0.3
Median thrombocyte engraftment duration (days)	12 (9–27)	14 (10–84)	0.007

## Discussion

Total body irradiation has an important role in the conditioning regimen for hematological malignancies such as acute leukemia and lymphomas. Especially, ALL is considered the ideal hematologic malignancy for which TBI-based conditioning for allo-SCT. In studies on TBI-based conditioning regimens, most evaluations were made on long-term transplantation results [13]. In the current study, we examined the effect of a transplantation conditioning regimen on bone marrow engraftment duration in a shorter time for Allo-SCT. We observed that thrombocyte engraftment occurred earlier than neutrophil engraftment in patients who received TBI-based conditioning, especially ALL patients. TBI-based conditioning regimens had a favorable impact on the recipient’s bone marrow microenvironment and precursor cells in the bone marrow, especially megakaryocytes, thus early thrombocyte engraftment occurred. However, myeloid cytotoxic conditioning regimens or TBI-based treatment regimens had a similar effect on neutrophil engraftment in Allo-SCT. The reason that TBI had an impact on the engraftment of thrombocytes but not neutrophils may be attributed to immune mechanisms which affect more TE than NE as TBI suppresses recipient-side immunity.

TBI has many advantages such as a uniform effect on the whole body and causes decreased exposure to cytotoxic chemotherapeutic regimens. To deplete patients’ HSCs in BM, ionizing irradiation has been used in Allo-SCT [18,19]. TBI damages the DNA of cells and inhibits their proliferation [20,21]. The TBI reduces tumor burden and provides an immunosuppressive effect and depletes the BM to allow enough space for the engraftment of healthy donor cells. The immunosuppressive effect of TBI on the host immune system minimizes the risk of engraftment failure [22-24]. A high dose of TBI has been reported to cause organ toxicities [11,16,25-28]. Because of this, researchers have tried strategies, including non-TBI-based or reduced-intensity conditioning (RIC) regimens. In previous studies, because of insufficient immunosuppression, non-TBI-based conditioning regimens or RIC were found to be associated with a higher risk of graft failure in Allo-SCTs which were performed from unrelated or HLA-mismatched donors [29-32]. Contrary to this; in the study conducted by Nakasone et al. in unrelated Allo-SCT cohorts, if an HLA-mismatched donor was selected, TBI did not have a favorable impact on NE [16]. In the current study, we evaluated only transplants from matched-related donors, excluding the negative effects of unrelated donors or haploidentical transplants.

In a previous study, a high-TBI-myeloablative conditioning regimen was found to be associated with delayed NE in the matched unrelated cohort and this was attributed to the damage of the BM environment due to high dose [33]. In our study, no statistically significant difference was observed between AL patients who received non-TBI-based conditioning or TBI-based conditioning regarding NE duration. On the other hand, TE duration was statistically significantly shorter in AL patients who received TBI-based conditioning when compared to those who received non-TBI-based conditioning. Patients are mostly supported by granulocyte colony-stimulating factor (G-CSF) agents for the shortened neutrophil engraftment duration, however, there is no standardized supportive approach for the delayed thrombocyte engraftment except thrombocyte transfusion. It is important to note that delayed thrombocyte engraftment has shown a risk factor for higher non-relapse mortality and worse overall survival [34].

Due to the retrospective and single-center design of the study, our study has some limitations. One of the study limitations is the limited number of patients recruited in our study. The other conditioning regimens were not standardized in the groups. We focused on the acute effects as engraftment duration mostly after Allo-SCT, we did not evaluate long-term graft failure. Nevertheless, the effect on engraftment duration with TBI-based conditioning is very limited in the literature. We concluded that TBI-based conditioning could provide a shorter thrombocyte engraftment duration than neutrophil. 

In summary, we found that TBI had a favorable impact on TE but not NE in matched related Allo-SCT. TBI-based conditioning suppresses recipient-side immunity before transplantation. The reason that TBI had an impact on the engraftment of thrombocytes compared to neutrophils may be attributed to immune mechanisms and microenvironment in the patient’s bone marrow.

## Conclusions

We analyzed the effect of TBI on neutrophil and thrombocyte engraftment durations in acute leukemia patients who were performed Allo-SCT. The conclusion that TBI had a favorable impact on thrombocyte engraftment rather than neutrophil engraftment in Allo-SCT in patients with acute leukemia, especially ALL patients, be supported by future multicenter studies with more patients.
